# Clinical Differences between Community-Acquired *Mycoplasma pneumoniae* Pneumonia and COVID-19 Pneumonia

**DOI:** 10.3390/jcm11040964

**Published:** 2022-02-12

**Authors:** Naoyuki Miyashita, Yasushi Nakamori, Makoto Ogata, Naoki Fukuda, Akihisa Yamura, Yoshihisa Ishiura, Shosaku Nomura

**Affiliations:** 1 Division of Respiratory Medicine, Infectious Disease and Allergology, First Department of Internal Medicine, Kansai Medical University, 2-3-1 Shinmachi, Hirakata 573-1191, Osaka, Japan; ogatam@hirakata.kmu.ac.jp (M.O.); fukudana@hirakata.kmu.ac.jp (N.F.); yamuraak@hirakata.kmu.ac.jp (A.Y.); nomurash@hirakata.kmu.ac.jp (S.N.); 2 Department of Emergency Medicine, Kansai Medical University Medical Center, 10-15 Fumizonocho, Moriguchi 570-8507, Osaka, Japan; 99nakamori@gmail.com; 3 Division of Respiratory Medicine, Oncology and Allergology, First Department of Internal Medicine, Kansai Medical University Medical Center, 10-15 Fumizonocho, Moriguchi 570-8507, Osaka, Japan; ishiuray@takii.kmu.ac.jp

**Keywords:** clinical differences, community-acquired pneumonia, *Mycoplasma pneumoniae*, SARS-CoV-2, COVID-19

## Abstract

*Mycoplasmapneumoniae* is one of the major causative pathogens of community-acquired pneumonia (CAP). *M. pneumoniae* CAP is clinically and radiologically distinct from bacterial CAPs. One feature of the Japanese Respiratory Society (JRS) guidelines is a trial to be carried out to differentiate between *M. pneumoniae* pneumonia and bacterial pneumonia for the selection of antibiotics. The purpose of the present study was to clarify the clinical and radiological differences of the *M. pneumoniae* CAP and coronavirus disease 2019 (COVID-19) CAP. This study was conducted at 5 institutions and assessed a total of 210 patients with *M. pneumoniae* CAP and 956 patients with COVID-19 CAP. The median age was significantly younger in patients with *M. pneumoniae* CAP than COVID-19 CAP. Among the clinical symptoms, cough and sputum were observed more frequently in patients with *M. pneumoniae* CAP than those with COVID-19 CAP. However, the diagnostic specificity of these findings was low. In contrast, loss of taste and anosmia were observed in patients with COVID-19 CAP but not observed in those with *M. pneumoniae* CAP. Bronchial wall thickening and nodules (tree-in-bud and centrilobular), which are chest computed tomography (CT) features of *M. pneumoniae* CAP, were rarely observed in patients with COVID-19 CAP. Our results demonstrated that there were two specific differences between *M. pneumoniae* CAP and COVID-19 CAP: (1) the presence of loss of taste and/or anosmia and (2) chest CT findings.

## 1. Introduction

*Mycoplasmapneumoniae* is one of the major causative pathogens of community-acquired pneumonia (CAP) and the most frequent pathogen in atypical pneumonia [1]. Epidemiological studies in Japan have demonstrated that the incidence of *M. pneumoniae* pneumonia is the second-to-third leading pathogen of CAP, accounting for as many as 10–30% of all cases of CAP [2,3,4]. Although pneumonia due to *M. pneumoniae* is usually of mild-to-moderate severity, some cases are known to develop into severe, life-threatening pneumonia [1,2,3,4]. Underlying conditions, clinical symptoms, laboratory data, and radiologic findings of *M. pneumoniae* pneumonia are different from other bacterial pneumonia [5,6]. Thus, the Japanese Respiratory Society (JRS) pneumonia guidelines proposed a differential diagnosis between other bacterial and *M. pneumoniae* pneumonia for the selection of an appropriate antibiotic for the management of CAP [2]. In addition, clinical findings of *M. pneumoniae* pneumonia are clearly different from *Legionella* CAP [7].

Since 2020, the novel, severe, acute respiratory syndrome, coronavirus 2 (SARS-CoV-2), has become the most important pathogen in CAP [8]. The purpose of the present study was to clarify the clinical and radiological differences of *M. pneumoniae* CAP and coronavirus disease 2019 (COVID-19) CAP.

## 2. Subjects and Methods

### 2.1. Study Population

The present study was conducted at five institutions (Kansai Medical University Hospital, Kansai Medical University Medical Center, Kansai Medical University Kori Hospital, Kansai Medical University Kuzuha Hospital, and Kansai Medical University Temmabashi General Clinic) between January 2017 and December 2021. We enrolled adult patients consecutively diagnosed with CAP, defined in accordance with the JRS guidelines [2]. The diagnosis was based on clinical signs and symptoms (cough, fever, productive sputum, dyspnea, chest pain, or abnormal breath sounds) and radiographic pulmonary abnormalities that were at least segmental and were not as a result of pre-existing or other known causes. Exclusion criteria included the following: immunosuppressive illness (i.e., HIV positive, neutropenia secondary to chemotherapy, use of >20 mg/day prednisone or other immunosuppressive agents, and history of organ transplant); hospitalization in the preceding 90 days; residence in a nursing home or extended care facility; receiving regular endovascular treatment as an outpatient (dialysis, antibiotic therapy, chemotherapy, immunosuppressant therapy); and active tuberculosis. All cases of pneumonia occurring more than three days after hospitalization were considered nosocomial and were excluded.

*M. pneumoniae* was diagnosed using positive culture and/or real-time polymerase chain reaction (PCR) results from nasopharyngeal swab specimens and/or a four-fold rise in the antibody titer level between paired sera. COVID-19 was diagnosed with positive PCR results from sputum or nasopharyngeal swab specimens in accordance with the protocol recommended by the National Institute of Infectious Diseases, Japan.

The severity of pneumonia was evaluated using predictive rules via the A-DROP system (a 6-point scoring system) proposed by the JRS guidelines: age over 70 years in men and over 75 years in women, dehydration, respiratory failure, orientation disturbance, and low blood pressure [2]. Patients were stratified into four severity classes: 0 point = mild, 1 or 2 points = moderate, 3 points = severe, and 4 or 5 points = extremely severe. The time between the clinical onset of pneumonia (fever and/or other symptoms) and judgement of pneumonia severity ranged from 1 to 14 days (mean, 4.8 days) for COVID-19 pneumonia and from 1 to 10 days (mean, 5.2 days) for *M. pneumoniae* pneumonia. Pneumonia severity score was judged before any treatment against both *M. pneumoniae* and COVID-19 pneumonia.

High-resolution computed tomography (CT) was performed in all patients with 1-mm collimation at 10-mm intervals. Informed consent was obtained from all patients, and the study protocol was approved by the Ethics Committee of Kansai Medical University (approval number 2020319).

### 2.2. Statistical Analysis

Discrete variables are expressed as counts (percentages) and continuous variables as medians and interquartile ranges. Frequencies were compared using Fisher’s exact test. Between-group comparisons of normally distributed data were performed using Student’s *t*-test. Skewed data were compared using the Mann–Whitney *U* test.

## 3. Results

### 3.1. Analysis Patients

The data of a total of 210 patients with *M. pneumoniae* CAP and 956 patients with COVID-19 CAP were analyzed. Cases of pneumonia mixed with other microorganisms were excluded from the study. During the study period, other microbiological diagnosis was established in 398 patients. The most common pathogens were *Streptococcus pneumoniae*, found in 281 cases, followed by *Haemophilus influenzae* in 93 cases, *Moraxella catarrhalis* in 21 cases and *Staphylococcus aureus* in 16 cases. Dual pathogens were detected in 34 cases. Of the 956 patients with COVID-19 CAP, 260 had lineage B.1.1.7, also known as the Alpha variant, and 274 had lineage B.1.617, also known as the Delta variant.

### 3.2. Clinical Presentation of M. pneumoniae CAP and COVID-19 CAP

Table 1 shows the underlying conditions and clinical findings of patients in the *M. pneumoniae* CAP and COVID-19 CAP groups at the first examination. The median age was significantly younger in patients with *M. pneumoniae* CAP than those with COVID-19 CAP. Among comorbid illnesses at baseline, the frequency of diabetes mellitus was significantly higher in patients with COVID-19 CAP than those with *M. pneumoniae* CAP.

Respiratory symptoms such as cough, sputum production, sore throat and chest pain were observed more frequently in patients with *M. pneumoniae* CAP than those with COVID-19 CAP. Cough is usually stubborn in *M. pneumoniae* CAP, but not in COVID-19 CAP. In contrast, loss of taste and anosmia were observed in patients with COVID-19 CAP, but not observed in those with *M. pneumoniae* CAP. Interestingly, the presence of runny nose was low frequency in both *M. pneumoniae* CAP and COVID-19 CAP.

With regard to pneumonia severity, approximately 90% of cases were mild-to-moderate severity in both CAP groups. The number of patients admitted to the intensive care unit and patients with in-hospital mortality were higher in patients with COVID-19 CAP than *M. pneumoniae* CAP. These findings were caused by the lack of a specific antimicrobial agent against COVID-19.

Of the 210 patients with *M. pneumoniae* CAP, 84 patients received minocycline, 64 patients received macrolides and 62 patients received quinolones. Eighteen severe patients received glucocorticoid in addition to antibiotics. Of the 956 patients with COVID-19 CAP, 648 patients received antiviral therapy, 258 patients received antibiotics and 619 patients received glucocorticoid.

### 3.3. Clinical Presentation of M. pneumoniae CAP and Age- and Gender-Matched COVID-19 CAP

Table 2 shows the underlying conditions and clinical findings of patients in the *M. pneumoniae* CAP and age- and gender-matched patients with COVID-19 CAP at the first examination. Among clinical symptoms, cough and sputum were observed more frequently in patients with *M. pneumoniae* CAP than those with COVID-19 CAP. In addition, loss of taste and anosmia were observed in one-third of patients with COVID-19 CAP.

Although pneumonia severity was identical between the unmatched COVID-19 CAP group and matched COVID-19 CAP group, the number of patients admitted to intensive care units and patients with in-hospital mortality were significantly reduced in patients with the matched COVID-19 CAP group.

### 3.4. Chest CT Findings

**Figure 1:**
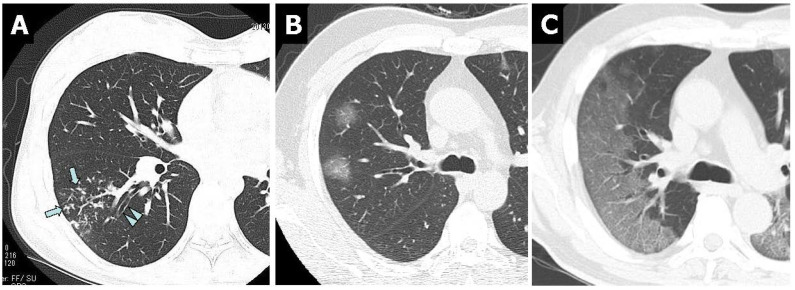
Non-contrast-enhanced thin-section axial images of the lungs in patients with *M. pneumoniae* pneumonia (**A**) and COVID-19 pneumonia (**B**,**C**). (**A**) Chest CT scan of a 46-year-old female showed nodules (tree-in-bud, arrows) and bronchial wall thickening (arrowheads). (**B**) Chest CT scan of a 46-year-old man showed bilateral and multifocal rounded GGO. (**C**) Chest CT in a 56-year-old man showed bilateral and peripheral GGO with superimposed interlobular septal thickening and crazy-paving appearance.

At the first CT examination within 10 days after symptom onset, bronchial wall thickening (83.8%) was observed most frequently, followed by nodules (tree-in-bud and centrilobular) (81.4%) in patients with *M. pneumoniae* CAP. In contrast to *M. pneumoniae* CAP, bronchial wall thickening and nodules (tree-in-bud and centrilobular) were rarely observed in patients with COVID-19 CAP (Figure 1).

## 4. Discussion

Patients with *M. pneumoniae* CAP have several distinct clinical features compared to patients with CAP due to other pathogens. *M. pneumoniae* infection occurs predominantly in school-aged children and younger adults. Cough is the main symptom, which is usually paroxysmal and often persistent. Peripheral white blood cell (WBC) count is usually normal at less than 10,000/µL. Thus, the JRS extracted six parameters from patients with *M. pneumoniae* pneumonia using multiple regression analysis [5]. Several studies have supported the usefulness of the JRS scoring system for distinguishing between *M. pneumoniae* pneumonia and other bacterial pneumonia [9,10]. In the present study, the sensitivity rates for presumptive diagnosis of *M. pneumoniae* CAP and COVID-19 CAP were 86.2% and 69.9%, respectively, based on four or more parameters of the criteria. The matching rates in two parameters, (1) age <60 years and (2) presence of stubborn cough, were significantly lower in COVID-19 CAP than in *M. pneumoniae* CAP. When the age- and gender-matched groups, only one parameter, the frequency of stubborn cough, was different between the two groups.

To increase the diagnostic sensitivity, in addition to the JRS scoring system, chest CT findings were a useful tool as an auxiliary diagnostic test to differentiate between *M. pneumoniae* CAP and bacterial CAP for the selection of antibiotics [11,12]. Typical chest CT findings of *M. pneumoniae* pneumonia resemble a combination of bronchial wall thickening and tree-in-bud and centrilobular nodules and/or ground-glass opacity with lobular distribution [11,12]. In contrast, bronchial wall thickening and nodules (tree-in-bud and centrilobular) were rarely observed in patients with COVID-19 CAP. These findings of chest CT among patients with COVID-19 pneumonia were consistent with previous reports [13,14,15]. Thus, physicians can differentiate *M. pneumoniae* CAP from COVID-19 CAP using chest CT findings.

A similar investigation was performed in pediatric patients using 80 patients with COVID-19 and 95 patients with *M. pneumoniae* CAP by Guo et al. [16]. Compared to COVID-19 and *M. pneumoniae*, fever and cough are observed more common in *M. pneumoniae* CAP. COVID-19 patients presented remarkably increased alanine aminotransferase. The typical CT feature of COVID-19 was ground-glass opacity, and it was more common in COVID-19 patients. Our results of clinical symptoms are similar to Guo’s results, but not similar in laboratory data and CT findings. These differences may be caused by adults and children.

In conclusion, we found several differences between *M. pneumoniae* CAP and COVID-19 CAP. *M. pneumoniae* CAP is significantly more common in younger patients, and the average age of patients with COVID-19 CAP is higher than that of patients with *M. pneumoniae* CAP. Cough, especially stubborn cough, and sputum production were more frequently observed in patients with *M. pneumoniae* CAP. However, the diagnostic specificity of these findings was low. The specific differences between *M. pneumoniae* CAP and COVID-19 CAP were (1) the presence of loss of taste and/or anosmia and (2) chest CT findings.

## Figures and Tables

**Table 1:** Underlying conditions and clinical findings in patients with *Mycoplasma pneumoniae* pneumonia and COVID-19 pneumonia at the first examination.

Variables	*M. pneumoniae*	COVID-19	*p*-Value
No. of patients	210	956	
Median age (IQR), years	36 (27–52)	56 (42–70)	<0.001
No. of males/females	106/104	599/357	0.001
No. (%) of patients with comorbid illnesses			
Chronic lung disease	19 (9.0)	107 (11.2)	0.393
Diabetes mellitus	11 (5.2)	167 (17.5)	<0.001
Chronic heart disease	6 (2.9)	45 (4.7)	0.269
Chronic liver disease	4 (1.9)	24 (2.5)	0.804
Cerebrovascular disease	3 (1.4)	26 (2.7)	0.338
Chronic renal disease	3 (1.4)	28 (2.9)	0.341
Neoplastic disease	2 (1.0)	30 (3.1)	0.100
Autoimmune disease	2 (1.0)	23 (2.4)	0.290
No. (%) of patients with the following clinical signs and symptoms			
Fever (≥37.0 °C)	207 (98.6)	822 (85.9)	<0.001
Cough	206 (98.1)	604 (63.2)	<0.001
Sputum production	126 (60.0)	126 (13.2)	<0.001
Sore throat	74 (35.2)	228 (23.8)	0.001
Headache	62 (29.5)	121 (12.7)	<0.001
Shortness of breath	32 (15.2)	293 (30.6)	<0.001
Chest pain	32 (15.2)	27 (2.8)	<0.001
Nausea or vomiting	17 (8.1)	22 (2.3)	0.0001
Runny nose	15 (7.1)	69 (7.2)	>0.999
Joint pain	14 (6.7)	58 (6.1)	0.752
Muscle ache	9 (4.3)	30 (3.1)	0.398
Diarrhea	5 (2.4)	82 (8.6)	0.001
Abdominal pain	2 (1.0)	22 (2.3)	0.288
Loss of taste	0	184 (19.2)	<0.001
Anosmia	0	167 (17.5)	<0.001
Laboratory findings, median (IQR)			
White blood cell count,/µL	6150 (5070–8460)	5200 (4200–6700)	0.122
C-reactive protein, mg/dL	6.4 (3.3–11.5)	4.4 (1.6–9.3)	0.241
Aspartate aminotransferase, U/L	29 (21–40)	34 (23–52)	0.126
Alanine aminotransferase, U/L	26 (20–39)	26 (18–43)	>0.999
No. (%) of patients with each pneumonia severity score			
Mild to moderate	192 (91.4)	865 (90.5)	0.793
Severe	16 (7.6)	76 (7.9)	>0.999
Extremely severe	2 (1.0)	15 (1.6)	0.752
No. (%) of patients admitted to intensive care unit	5 (2.4)	290 (30.3)	<0.001
No. (%) of patients with in-hospital mortality	0	18 (1.9)	0.057

Continuous values are presented as medians and interquartile ranges and categorical/binary values as counts and percentages. IQR: interquartile ranges.

**Table 2:** Underlying conditions and clinical findings in patients with *Mycoplasma pneumoniae* pneumonia and age and gender matched COVID-19 pneumonia at the first examination.

Variables	*M. pneumoniae*	COVID-19	*p*-Value
No. of patients	210	210	
Median age (IQR), years	36 (27–52)	36 (27–52)	>0.999
No. of males/females	106/104	106/104	>0.999
No. (%) of patients with comorbid illnesses			
Chronic lung disease	19 (9.0)	15 (7.1)	0.592
Diabetes mellitus	11 (5.2)	21 (10.0)	0.097
Chronic heart disease	6 (2.9)	4 (1.9)	0.751
Chronic liver disease	4 (1.9)	3 (1.4)	>0.999
Cerebrovascular disease	3 (1.4)	3 (1.4)	>0.999
Chronic renal disease	3 (1.4)	2 (1.0)	>0.999
Neoplastic disease	2 (1.0)	2 (1.0)	>0.999
Autoimmune disease	2 (1.0)	2 (1.0)	>0.999
No. (%) of patients with the following clinical signs and symptoms			
Fever (≥37.0 °C)	207 (98.6)	178 (84.8)	<0.001
Cough	206 (98.1)	141 (67.1)	<0.001
Sputum production	126 (60.0)	29 (13.8)	<0.001
Sore throat	74 (35.2)	73 (34.8)	>0.999
Headache	62 (29.5)	46 (21.9)	0.094
Shortness of breath	32 (15.2)	42 (20.0)	0.249
Chest pain	32 (15.2)	9 (4.3)	0.0002
Nausea or vomiting	17 (8.1)	7 (3.3)	0.057
Runny nose	15 (7.1)	17 (8.1)	0.854
Joint pain	14 (6.7)	26 (12.4)	0.066
Muscle ache	9 (4.3)	12 (5.7)	0.655
Diarrhea	5 (2.4)	14 (6.7)	0.058
Abdominal pain	2 (1.0)	7 (3.3)	0.175
Loss of taste	0	68 (32.4)	<0.001
Anosmia	0	70 (33.3)	<0.001
Laboratory findings, median (IQR)			
White blood cell count,/µL	6150 (5070–8460)	5500 (4400–6200)	0.208
C-reactive protein, mg/dL	6.4 (3.3–11.5)	4.3 (1.8–9.0)	0.299
Aspartate aminotransferase, U/L	29 (21–40)	29 (22–46)	>0.999
Alanine aminotransferase, U/L	26 (20–39)	25 (19–41)	0.891
No. (%) of patients with each pneumonia severity score			
Mild to moderate	192 (91.4)	204 (97.1)	0.594
Severe	16 (7.6)	6 (2.9)	0.046
Extremely severe	2 (1.0)	0	0.499
No. (%) of patients admitted to intensive care unit	5 (2.4)	18 (8.6)	0.009
No. (%) of patients with in-hospital mortality	0	0	>0.999

Continuous values are presented as medians and interquartile ranges (IQRs) and categorical/binary values as counts and percentages. IQR: interquartile ranges.
